# A new *in vitro* hemagglutinin inhibitor screening system based on a single-vesicle fusion assay

**DOI:** 10.1038/srep30642

**Published:** 2016-07-29

**Authors:** Hanki Lee, Wook Jin, Byeong-Chul Jeong, Joo-Won Suh

**Affiliations:** 1 Center for Nutraceutical and Pharmaceutical Materials, Myongji University, Yongin, Gyeonggi-do, 17058, Republic of Korea; 2 Laboratory of Molecular Disease and Cell Regulation, Department of Molecular Medicine, School of Medicine, Gacheon University, Incheon, 21936, Republic of Korea; 3 Gachon Medical Research Institute, Gil Medical Center, Incheon, 21565, Republic of Korea; 4 Division of Biosciences and Bioinformatics, College of Natural Science, Myongji University, Yongin, Gyeonggi-do, 17058, Republic of Korea

## Abstract

Hemagglutinin (HA) from the influenza virus plays a pivotal role in the infection of host mammalian cells and is, therefore, a druggable target, similar to neuraminidase. However, research involving the influenza virus must be conducted in facilities certified at or above Biosafety Level 2 because of the potential threat of the contagiousness of this virus. To develop a new HA inhibitor screening system without intact influenza virus, we conceived a single-vesicle fusion assay using full-length recombinant HA. In this study, we first showed that full-length recombinant HA can mediate membrane fusion in ensemble and single-vesicle fusion assays. The fluorescence resonance energy transfer (FRET) frequency pattern of single-vesicle complexes completely differed when the inhibitors targeted the HA1 or HA2 domain of HA. This result indicates that analysing the FRET patterns in this assay can provide information regarding the domains of HA inhibited by compounds and compounds’ inhibitory activities. Therefore, our results suggest that the assay developed here is a promising tool for the discovery of anti-influenza virus drug candidates as a new *in vitro* inhibitor screening system against HA from the influenza virus.

Specific binding to the host cell membrane and the exposure of the viral genetic material in the host cytoplasm are important steps in the process of viral infection^1^,^2^. In the influenza virus, hemagglutinin (HA), a glycoprotein found in the viral envelope, is responsible for both the specific binding to the host cell membrane and the release of the viral genome from the late endosomes of the host cells upon membrane fusion^1^,^2^. To date, 16 subtypes (H1–H16) of HA have been identified, and the type of HA present is a major factor in host infection^3^,^4^,^5^. HA from the influenza virus is divided into two functional domains: HA1 and HA2. HA1 accelerates the binding of the virus by recognising sialic acids on the host membrane, whereas HA2 mainly participates in membrane fusion through a conformational change within its N-terminal fusogenic peptide at low pH^2^.

Five drugs have been approved by the U.S. Food and Drug Administration (FDA) to treat the influenza virus^6^. Amantadine and rimantadine target the M2 proton channel of the influenza virus and inhibit proton translocation through this channel after the influenza virus enters host cells via endocytosis^6^. However, influenza viruses resistant to these two drugs have emerged; therefore, the FDA no longer recommends these drugs for the treatment of this virus^6^. The three remaining drugs—zanamivir, oseltamivir phosphate and peramivir—target neuroaminidase (NA) from the influenza virus and inhibit the detachment of this virus from the host membrane after the assembly of viral molecules in the cytoplasm^6^. Although these three drugs are very effective and are used to treat the influenza virus, viruses resistant to these drugs have been discovered^7^. Thus, the emergence of influenza viruses that are resistant to existing drugs has reinforced the need to develop anti-influenza virus drug candidates with new targets.

As mentioned above, membrane fusion by HA from the influenza virus is a key process in this virus’s life cycle; therefore, the discovery of specific inhibitors of HA could represent a new strategy to develop anti-influenza virus drugs. However, research using influenza virus can only be performed in facilities certified at or above Biosafety Level 2 because of the potential threat of this virus. To overcome this limitation, in this study, we developed a new, threat-free *in vitro* influenza virus fusion assay and examined its use as an *in vitro* HA inhibitor screening system.

## Materials and Methods

### Preparation of single vesicles for the ensemble and single-vesicle fusion assays

To prepare single vesicles in this study, we selected lipids to design membranes with compositions similar to that of the influenza viral membrane. We chose the four lipids based on previous research: phosphatidylserine, phosphatidylcholine, phosphatidylethanolamine, and cholesterol^8^,^9^,^10^. The lipids used in this study were 1,2-dioleoyl-sn-glycero-3-phospho-L-serine (DOPS), 1-palmitoyl-2-oleoyl-sn-glycero-3-phosphocholine (POPC), 1,2-dioleoyl-sn-glycero-3-phosphoethanolamine (DOPE), cholesterol, a total ganglioside extract (the sialic acid component), 1,2-dipalmitoyl-sn-glycerol-3-phosphoethanolamin-N-(biotinyl) (biotin-DPPE), 1,1′-dioctadecyl-3,3,3′,3′-tetramethylindocarbocyanine perchlorate (DiI, Invitrogen) and 1,1′-dioctadecyl-3,3,3′,3′-tetramethylindodicarbocyanine perchlorate (DiD, Invitrogen). All lipids except for DiI and DiD were purchased from Avanti Polar Lipids. HA was purchased from Abcam (Cat. No. ab69741), and its subtype was H1. The molar ratios of the lipid species in the HA-containing vesicles (H-vesicles) and sialic acid-containing vesicles (S-vesicles) were 20.2:38:25:15:1.8 (DOPS:POPC:DOPE:cholesterol:DiI) and 19.8:38:25:15:0.1:0.1:2 (DOPS:POPC:DOPE:cholesterol:total ganglioside extract:biotin-DPPE:DiD), respectively. Vesicles were prepared by a direct method^11^,^12^. Briefly, to prepare the H-vesicles, the lipids were mixed in a glass vial at the desired ratio and then completely dried to remove the organic solvents. The dried lipid mixture was hydrated using a reaction buffer (20 mM 4-(2-hydroxyethyl)-1-piperazineethanesulfonic acid [HEPES], 0.1 M KCl and 20% glycerol, pH 7.5) containing 1% N-octyl-β-D-glucopyranoside (OG). HA was dissolved in the same buffer as the OG but in a separate tube. Next, HA and the hydrated lipid film were mixed at a ratio of 250:1, except for experiments in which various lipid-to-HA ratios were used. After incubation for 1 h at 4 °C with gentle shaking, the lipid-OG-HA mixture was rapidly diluted by more than 3-fold to lower the OG concentration to below the critical micelle concentration. This diluted mixture was then dialyzed in 1 L of reaction buffer at 4 °C overnight with 2 g of pre-cleaned SM-2 Biobeads (Bio-Rad) dissolved in reaction buffer. After dialysis, the H-vesicle solution was centrifuged at 10,000 × *g* for 30 min at 4 °C, and the supernatant was recovered. S-vesicles were prepared using the same procedure as for H-vesicles without HA. The hydrodynamic sizes of the H- and S-vesicles were determined by dynamic light scattering (Zetasizer Nanoseries ZS, Malvern Instruments). The distributions of the H- and S-vesicle sizes were measured at 25 °C after 5 min of equilibration.

To determine the ratio of HA incorporated into H-vesicles, a co-flotation assay was performed. Briefly, 400 μl of H-vesicles was mixed with 400 μl of 80% Histodenz (Sigma-Aldrich) in an ultracentrifuge tube and overlaid with 300 μl of 30% Histodenz followed by 100 μl of fusion buffer. H-vesicles and Histodenz were dissolved in the reaction buffer. After centrifuging the sample at 280,000 × *g* for 2.5 h in a SW50 Ti rotor (Beckman), 350 μl of the vesicles were collected from the 0%/30% Histodenz interface and concentrated to 50 μl using an Ultracel-3 K membrane (Millipore). The sample was analysed by 10% sodium dodecyl sulfate polyacrylamide gel electrophoresis (SDS-PAGE) followed by Coomassie brilliant blue staining.

### Ensemble assay for HA-driven fusion

H-vesicles and S-vesicles were added to a black 96-well plate, and the mixture was incubated for 5 min at room temperature. The fluorescence resonance energy transfer (FRET) level upon membrane fusion governed by HA was monitored by fluorescence spectroscopy with excitation at 549 nm and emission at 665 nm and a slit width of 10 nm in both cases. Full mixing of the H-vesicles and S-vesicles was defined using the fluorescence emission at 665 nm following the addition of Triton X-100 to a concentration of 1% at 300 sec. To monitor HA-mediated fusion by trypsin treatment, we added trypsin at 150 s and measured the fluorescence intensity at an emission wavelength of 665 nm until 300 s. The fusion efficiency in each experiment was determined by dividing the fluorescence intensity at 300 s by the maximum fluorescence intensity after the addition of Triton X-100.

### Imaging of single vesicle-vesicle events by total internal reflection fluorescence (TIRF) microscopy

Details of the method used are given by Lee *et al*. and Diao *et al*.^11^,^12^. The quartz slide was cleaned with piranha solution followed by 1 M potassium hydroxide and then coated with 99:1 (mol/mol) methoxypoly(ethylene glycol) (mPEG):biotin-PEG (Laysan Bio). The PEG-passivated quartz slide was used as the bottom surface of a microfluidic chamber for prism-type TIRF microscopy based on a IX-72 instrument (Olympus). To monitor the interaction between H- and S-vesicles, S-vesicles were immobilised on the quartz biotin-PEG imaging surface with Neutravidin (Invitrogen). To remove residual S-vesicles, the chamber was washed with reaction buffer, and the H-vesicles were loaded into the chamber. After incubation for 5 min, residual H-vesicles were removed by washing with reaction buffer. The number of S-vesicles was carefully controlled to be 712 ± 35. Therefore, the number of single-vesicle complexes in one imaging area could be directly compared for all experiments by the single-vesicle fusion assay in this study.

To monitor the fusion pattern following trypsin treatment, trypsin-containing reaction buffer was loaded into the chamber after removing the residual H-vesicles and incubated for 5 min. Thereafter, the buffer in the chamber was replaced with 50 mM citric acid buffer (pH 5.0). S-vesicles with and without sialic acid were prepared and immobilised individually, and then, the fusion patterns were observed using the procedure described above.

Single fusion events between H- and S-vesicles were measured in a wide-field TIRF microscope using an electron multiplying charge-coupled device (CCD) camera. Details of the wide-field TIRF microscope have been reported previously^13^. Briefly, an area of 45 × 90 μm^2^ was imaged using an inverted microscope (IX73, Olympus, Melville, NY) excited by a frequency-doubled Nd:yttrium aluminium garnet (YAG) laser (532 nm; Crystalaser, Reno, NV). The excitation beam was focused into a small pellin broca prism (CVI Laser, Albuquerque, NM), which was placed on top of a quartz slide separated by a thin layer of immersion oil to match the index of refraction. By changing the incident angle of the excitation beam, total internal reflection at the interface between the quartz slide and aqueous imaging buffer was achieved. The fluorescence signal was collected with a high NA water immersion objective (UPLAPO60XW; Olympus), and the scattered laser light was rejected by a 550-nm long-pass interference filter (E550LP; Chroma Technology, Rockingham, VT). A dichroic mirror with a reflection range of 550 to 630 nm (645DCXR; Chroma Technology) separated the collected fluorescence signal into two beams with different wavelengths: the donor (550–630 nm) and the acceptor (645 nm and above) channels. These two beams were focused on the electron-multiplying CCD camera (iXon DV 887-BI; Andor Technology, South Windsor, CT). The fluorescence signal was recorded in real time using Visual C software (Microsoft, Redmond, WA) (the program was written by Sean A. McKinney) with a time resolution of 100 ms. Each single fusion event was visually identified and analysed using programs written in IDL (Research Systems, Boulder, CO).

A program written in MATLAB (Mathworks, Natick, MA, USA) generated the time trajectories of the donor and acceptor fluorescence intensities and calculated the corresponding FRET efficiency using the following equation: *I*_*A*_/(*I*_*D*_ + *I*_*A*_), where *I*_*D*_ and *I*_*A*_ are the donor and acceptor fluorescence intensities, respectively. The average donor and acceptor fluorescence intensities measured before docking were considered to be the background fluorescence for each fusion event and subtracted uniformly from the fluorescence signals. The leakage of donor fluorescence into the acceptor channel (≈17.5% of the total intensity) was then accounted for. Intermediate states of the fusion event, which appeared as FRET efficiency plateaus, were visually identified, and the corresponding FRET efficiencies and dwell times were calculated using a program written in MATLAB.

To analyse the number of docked vesicles, an algorithm able to recognise the local Gaussian maxima in TIRF images recorded by an electron-multiplying CCD (iXon DU897v, Andor Technology) was used to count the number of single-vesicle complexes in field of view of 45 × 90 μm^2^ (available at https://cplc.illinois.edu/software/). Also, the quality of this single vesicle fusion assay suing HA was evaluated by for Z’ factor, which was calculated as follows:^14^


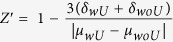


*δ*_*wU*_ and *δ*_*woU*_ indicate the standard deviation of the number of single vesicle complexes using H-vesicle after ultracentrifugation and without ultracentrifugation, respectively. And, *μ*_*wU*_ and *μ*_*woU*_ indicate the average of the number of single vesicle complexes using H-vesicle after ultracentrifugation and without ultracentrifugation, respectively.

For real-time experiments, movies of the field of view were recorded with a time resolution of 100 msec. To reduce fluorescence blinking, 50 mM citric acid buffer (pH 5.5) containing 1 mM Trolox (Sigma-Aldrich) was used. Using the same algorithm as for the docking number analysis, each vesicle-vesicle docking event and the subsequent fusion process were individually identified and tracked in the movie. The FRET efficiency was quantified using the following equation: *I*_*A*_/(*I*_*D*_ + *I*_*A*_), where *I*_*D*_ and *I*_*A*_ are the donor and acceptor fluorescence intensities, respectively. To quantitatively analyse the fusion kinetics, stepwise increases in the FRET efficiency were identified by a custom-written MATLAB (Mathworks) program that uses the Schwarz information criterion.

An anti-HA1 antibody (Cat. No. 10R-7995, Fizgerald, US), which was raised in mouse using recombinant human H1N1/HA1 (18-344aa) purified from Baculovirus as the immunogen, and tert-butylhydroquinone (TBHQ, Sigma) were used to inhibit the HA1 and HA2 domains of HA, respectively. These inhibitors were pre-incubated with the H-vesicles for 30 min with gentle shaking at 4 °C, and then, the inhibitor-H-vesicle mixture was loaded into the chamber after immobilising the S-vesicles on the imaging slide. The next steps were conducted as described above.

## Results

### Ensemble assay using full-length recombinant HA

**Figure 1:**
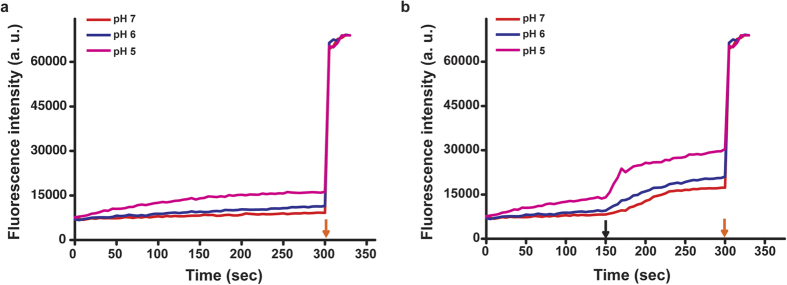
HA-driven fusion in the ensemble assay. (**a**) In the absence of trypsin treatment. (**b**) In the presence of trypsin treatment. The fluorescence intensity was monitored by measuring the emission at 665 nm. The trypsin concentration was 1%, which exceeded the amount required for the complete digestion of HA in H-vesicles. Black and orange arrows indicate the times at which trypsin and 1% Triton X-100 were added, respectively.

We first prepared full-length recombinant HA-containing vesicles labelled with DiI (H-vesicles) and sialic acid-containing vesicles labelled with DiD (S-vesicles), which mimic the influenza virus and host membrane, respectively. The sizes of the H- and S-vesicles were 43 and 40 nm, respectively, as determined by dynamic light scattering (Supplementary Fig. 1). HA was incorporated into the H-vesicles in a concentration-dependent manner (Supplementary Fig. 1, inset). To examine whether the incorporated HA could mediate membrane fusion, we performed an ensemble assay using H-vesicles, in which the lipid-to-HA ratio was 250:1, and S-vesicles. The fusion rate was below 18% at all tested pH values before the addition of trypsin relative to the fluorescence level observed upon full mixing after the addition of Triton X-100 (Fig. 1a). However, after trypsin treatment, the fusion rate increased in all tested pH conditions. Notably, the fusion rate immediately increased following the addition of trypsin and reached 43% at pH 5 (Fig. 1b). This result indicates that the incorporated HA could mediate membrane fusion and that the rate of HA-mediated fusion was accelerated by trypsin treatment and lower pH values (Fig. 1 and Supplementary Fig. 2). Interestingly, although trypsin was added, the fusion rate was not accelerated at pH 6 or 7, at which the fusion rates were 19% and 24%, respectively (Fig. 1b). Based on this result, we inferred that both exposure of the fusogenic peptide HA2 following trypsin treatment and a conformational change in HA2 at a lower pH are essential for HA-driven membrane fusion, in accordance with previous studies^15^,^16^,^17^.

### Single-vesicle fusion assay using full-length active HA

**Figure 2:**
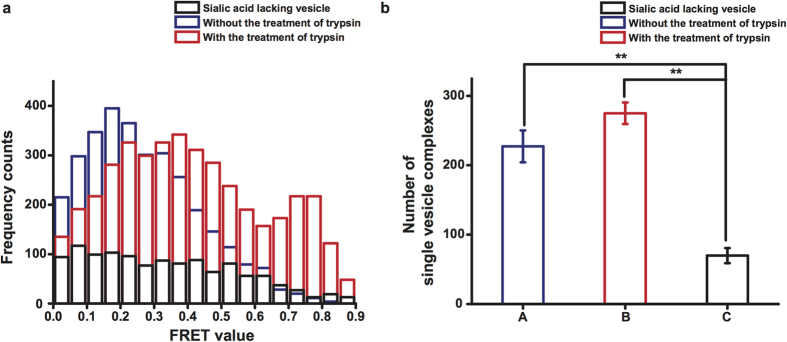
HA-driven fusion in the single-vesicle fusion assay. (**a**) Fusion pattern of single-vesicle complexes according to the interaction between the H- and S-vesicles. (**b**) The number of single-vesicle complexes according to the interaction between the H- and S-vesicles. This experiment was performed in triplicate. **P < 0.01, assessed using the paired t test. All errors indicate standard deviations.

**Figure 3:**
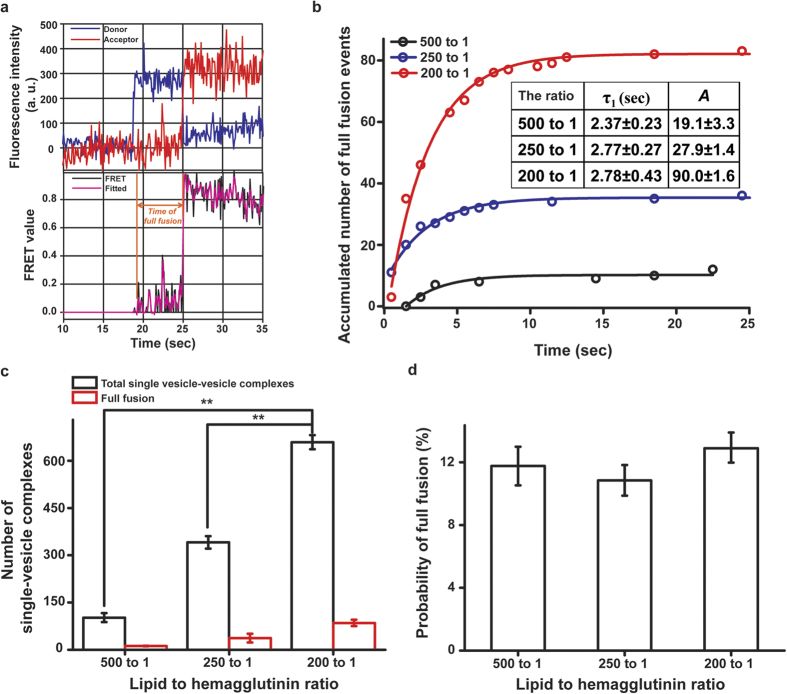
Analysis of HA-driven fusion in real-time experiments. (**a**) Exemplary real-time traces of full fusion events at the single-vesicle level. The top panel indicates the changes in the donor (blue, 565 nm emission) and acceptor (red, 665 nm emission) fluorescence intensities. The bottom panel presents the FRET (black) corresponding to the changes in the donor and acceptor fluorescence intensities shown in the top panel and the stepwise increases in FRET signals identified using the Schwarz information criterion (magenta). (**b**) The kinetics of HA-driven full fusion events with different lipid-to-HA ratios. The data were fit by a single exponential function. The inset table shows the time constants and populations fit by a single exponential function in each experiment. *A* indicates the fully fused population derived by fitting the data to a single exponential function. (**c**) The number of single-vesicle complexes formed between the H- and S-vesicles with different lipid-to-HA ratios. (**d**) The probability of HA-driven full fusion at different lipid-to-HA ratios. All experiments were conducted in triplicate. **P < 0.01, assessed using the paired t test. All errors indicate standard deviations.

To further investigate the results of the ensemble assay, we conducted a single-vesicle fusion assay with H- and S-vesicles in pH 5.0. First, we analysed the FRET patterns of single-vesicle complexes following trypsin treatment and in the presence of sialic acid as the specific ligand recognised by the HA1 domain of HA. In the absence of trypsin treatment, most vesicle complexes had a FRET value of 0.2–0.4, indicating a docked state. We can infer the state of single-vesicle complexes by analysing FRET values^11^,^12^,^18^,^19^. Therefore, we inferred that most single-vesicle complexes were in the docked state rather than a fully fused state in the absence of trypsin treatment. However, after trypsin treatment, the number of single-vesicle complexes in the fully fused state dramatically increased (Fig. 2a). Otherwise, the number of single-vesicle complexes was similar regardless of trypsin treatment (Fig. 2b). This result indicates that HA-driven docking and fusion are precisely governed by the HA1 and HA2 domains of this protein, respectively. We also monitored the fusion pattern using S-vesicles without sialic acid to determine how sialic acid affects HA-driven fusion. The total number of single-vesicle complexes in the fully fused and docked states was severely reduced (Fig. 2a,b). Thus, sialic acid promotes docking by HA and affects the fusion of single-vesicle complexes docked because of the specific binding of HA to sialic acid. To confirm that this fusion is specifically mediated by HA at low pH, we monitored the fusion patterns of H-vesicles without HA and S-vesicles under various pH conditions and trypsin treatment. H-vesicles without HA did not trigger fusion with S-vesicles at any pH, despite trypsin treatment (Supplementary Fig. 3). Also, in this assay, it is an important factor to get HA containing proteoliposome so we analyzed the number of single vesicle complexes and the Z’ factor using H-vesicle in accordance with ultracentrifugation, which is the step of harvest of H-vesicle. As a result, in the case of H-vesicle after ultracentrifugation, the average of number of single vesicle complexes was calculated to be 314.16. Meanwhile, in the case of H-vesicle without ultracentrifugation, the number was 74.41. And, Z’ factor, which determines the robustness of assay, was calculated to be 0.60 in this assay (Supplementary Fig. 4). Next, to monitor the fusion kinetics in terms of the lipid-to-HA ratio at the single-vesicle level, we conducted a real-time experiments using S-vesicles and H-vesicles containing different concentrations of HA. In the absence of trypsin, most HA vesicles remained in the docked state, in accordance with a batch experiment (data not shown); however, trypsin treatment quickly triggered full fusion between the H- and S-vesicles, in good agreement with a batch experiment (Fig. 3). This real-time experiment exclusively determined both the population of single-vesicle complexes and the timing of individual full fusion events in the single-vesicle fusion assay separate from the docking kinetics^12^. The time at which full fusion was reached was determined by fitting the FRET signal with the Schwarz information criterion, with FRET values of 0.2 and 0.7 corresponding to the docked and fully fused states, respectively (Fig. 3a). The time at which full fusion was achieved exhibited a single exponential distribution, regardless of the concentration of HA incorporated into the H-vesicles (Fig. 3b). The time constants for fusion were 2.37, 2.77 and 2.78 sec for lipid-to-HA ratios of 500:1, 250:1 and 200:1, respectively (Fig. 3b and inset). Interestingly, the number of single-vesicle complexes in both the docked and fully fused states increased in a HA concentration-dependent manner; however, the probability of full fusion was not influenced by the concentration of HA incorporated into the H-vesicles (Fig. 3c,d). These results showed that docking was rapidly enhanced by the number of HA1 domains incorporated into the H-vesicles and that fusion may be governed by an active HA2 domain.

### Application of the new *in vitro* HA inhibitor screening assay

**Figure 4:**
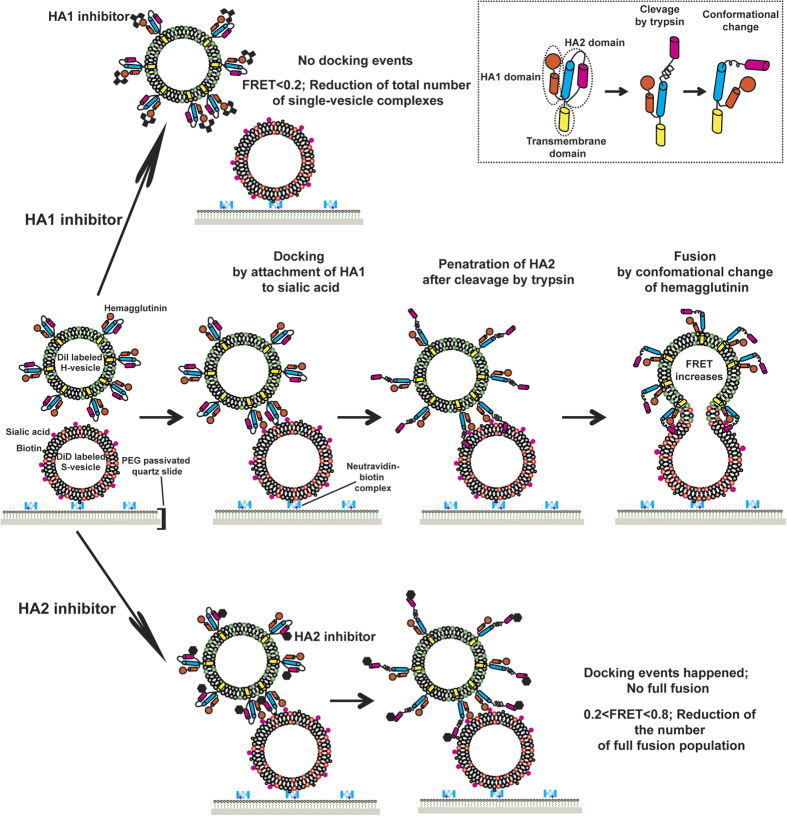
A schematic of HA-driven fusion and HA inhibition by inhibitors. DiI-labelled H-vesicles attach by specific docking of the HA1 domain to sialic acid in S-vesicles immobilised by the biotin-Neutravidin interaction on PEG-passivated quartz slides. Thereafter, exposure of the fusogenic peptide HA2 upon trypsin treatment and a conformational change of this domain can trigger membrane fusion between the H- and S-vesicles. When the DiI and DiD lipophilic dyes, which are the donor and acceptor fluorescent dyes, respectively, are mixed, the FRET value increases (middle panel). If the HA1 inhibitor binds to the HA1 domain, docking by HA no longer occurs, and therefore, the number of single-vesicle complexes between the H- and S-vesicles should decrease (upper panel). If the HA2 inhibitor specifically interacts with the HA2 domain, docking by HA occurs, but full fusion caused by a conformational change in HA2 should decrease. Therefore, after HA2 inhibitor treatment, most single-vesicle complexes will be in the docked or hemifusion state, corresponding to FRET values of 0.2–0.8. Inset indicates the conformational change of HA resulting from trypsin treatment at lower pH.

**Figure 5:**
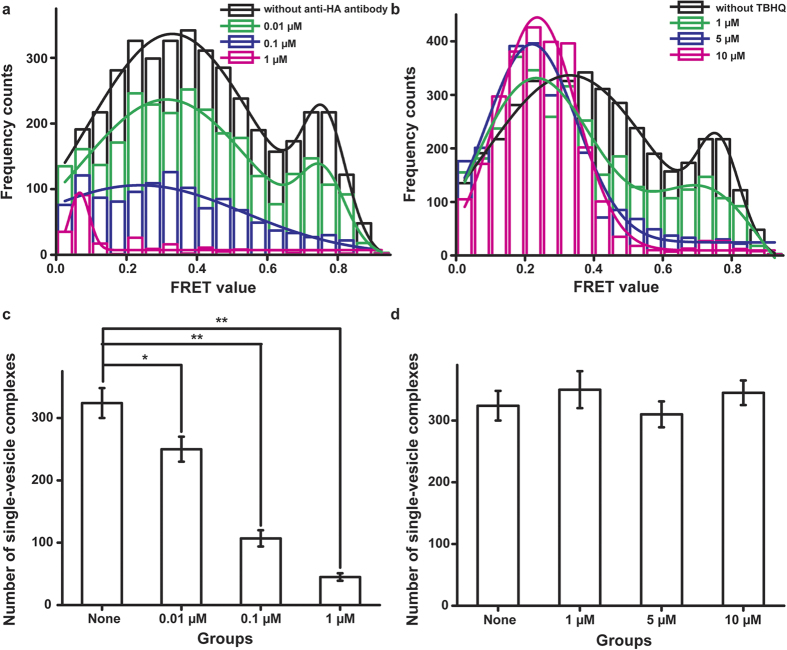
Analysis of the FRET patterns of single-vesicle complexes formed between H- and S-vesicles following treatment with inhibitors. (**a**) Treatment with an anti-HA1 antibody. The data obtained following no treatment (black) and treatment with 0.01 μM antibody were fit by multiple peak analysis based on a Gaussian algorithm. The data obtained following the two other treatments were fit using a Gaussian algorithm. (**b**) Treatment with TBHQ. The data obtained following no treatment (black) and treatment with 1 μM TBHQ were fit by multiple peak analysis based on a Gaussian algorithm. The data obtained following the two other treatments were fit using a Gaussian algorithm. (**c**) The number of single-vesicle complexes formed between the H- and S-vesicles with different anti-HA1 antibody concentrations. (**d**) The number of single-vesicle complexes formed between H- and S-vesicles with different TBHQ concentrations. All experiments were conducted in triplicate. ***P < 0.05 and **P < 0.01, assessed using the paired t test. All errors indicate standard deviations.

Based on previous batch and real-time experiments, we applied this assay to screen for inhibitors targeting HA and thereby discover anti-influenza virus drug candidates. If inhibitors can specifically bind to the HA1 domain, the population of single-vesicle complexes in the docked state should decrease. Additionally, if inhibitors can specifically bind to the HA2 domain, the population of single-vesicle complexes in the fully fused state should decrease (Fig. 4). As a “proof-of-concept” for the use of this method to screen HA inhibitors, we treated H-vesicles with an anti-influenza A HA1 antibody to neutralise the HA1 domain and with the HA2 inhibitor TBHQ^20^,^21^,^22^. We then analysed the fusion patterns in a batch experiment. Following treatment with the anti-HA1 antibody, the number of single-vesicle complexes in both the docked and fused states decreased in an antibody concentration-dependent manner relative to the negative control, in which vesicles were not treated with this antibody (Fig. 5a,c). Notably, when the vesicles were treated with 1 μM antibody, the interaction between the H- and S-vesicles completely disappeared. Prior to the experiment with TBHQ, we directly treated immobilised S-vesicles with various concentrations of dimethyl sulfoxide (DMSO) and then monitored the number of S-vesicles to ensure that the DMSO concentration did not affect them. The fluorescence distribution of the immobilised S-vesicles was not altered by treatment with up to 1% DMSO. However, it was severely changed, and large vesicle aggregates appeared following treatment with 5% DMSO, which destroyed the S-vesicles (Supplementary Fig. 5). Therefore, to avoid the destruction of the H- and S-vesicles by DMSO, we ensured that the final DMSO concentration was 1% when the H-vesicles were treated with TBHQ. Following TBHQ treatment, the population of docked single vesicles increased, whereas the fully fused population decreased in a TBHQ concentration-dependent manner (Fig. 5b). Interestingly, the number of single-vesicle complexes did not change regardless of the TBHQ concentration (Fig. 5d). Based on these results, we concluded that the change in the fusion pattern mediated by HA was related to inhibitors targeting the HA1 or HA2 domain of this protein in this screening assay. Therefore, this screening assay can be used to obtain information regarding the inhibitor-targeted HA domain by analysing fusion patterns.

## Discussion

In this study, we showed that full-length recombinant HA can trigger single-vesicle fusion at low pH values and upon treatment with trypsin. This assay basically monitors the interaction of H-vesicle with S-vesicle so the preparation of HA containing proteoliposomes is an essential step. Therefore, we analysed Z’ factor by H-vesicle according to the treatment of ultracentrifugation. As a result, the Z’ factor was 0.60 and it means that this assay is robust because the Z’ factor above 0.5 is considered as representative of a very robust assay (Supplementary Fig. 4)^14^. Therefore, this result indicates that the preparation of HA containing proteoliposomes by ultracentrifugation is responsible for the robustness of this assay. We also developed a new method to screen anti-HA inhibitors that does not require handling influenza virus. The single-vesicle assay reported here differs from those described previously in several important aspects. First, this assay can specifically monitor the entire HA-governed fusion process from docking to fusion using a simple FRET-based analysis of individual single-vesicle events (Figs 2 and 3). Some previous studies used fluorescently labelled influenza virus with supported membranes and tracked hemifusion and pore opening in the full fusion state^23^,^24^,^25^,^26^. However, these studies had difficulty observing influenza virus docking because the fluorescence level increased as a result of the de-quenching of R18, which is a lipophilic fluorescent dye, after the hemifusion of labelled influenza virus membranes. Even so, these methods can be used to quantitatively study pore opening by HA. One previous study used human red blood cells to mimic the host membrane instead of supported membranes and vesicles reconstituted with HA purified from influenza virus culture using egg phosphatidylcholine, cholesterol and R18 8. However, this method monitored the de-quenching of R18, and therefore, it has the same limitations as described above. Recently, another research group studied the influenza viral infection pathway through quantum dot-based single-particle tracking^27^,^28^. That group developed this technique for the long-term and real-time observation of influenza virus infection of host cells and dissected the infectious behaviours of H9N2 influenza viruses in individual cells. However, this technique focuses on tracking the movement of the influenza virus itself and is therefore not suitable to study the specific fusion process governed by HA. Second, to monitor HA-driven fusion, we first used full-length recombinant HA expressed in Sf9 insect cells and showed that it could mediate membrane fusion using the developed assay. Therefore, this assay can be widely used without any contagion threat arising from the direct handling of the influenza virus, as occurs during virus cultivation and the purification of HA from cultivated virus. Many research groups have used influenza virus itself or HA purified from cultivated virus^8^,^15^,^23^,^24^,^25^,^26^,^27^,^28^,^29^. The handling of influenza virus is restricted to facilities certified at or above Biosafety Level 2 to prevent the spread of influenza viruses. Therefore, research involving influenza virus must be performed under restrictions, even though directly using the influenza virus is experimentally advantageous. Third, we demonstrated that this assay constitutes a new *in vitro* HA inhibitor screening tool because of the use of an anti-HA1 antibody and TBHQ to inhibit HA1 and HA2, respectively (Fig. 5). Zanamivir, oseltamivir phosphate and peramivir, which specifically inhibit the NA activity of the influenza virus, are effective for the treatment of this virus; however, some resistant influenza viruses were recently discovered^7^,^30^. To address this issue, new druggable targets within the influenza virus and new, corresponding drug candidates must be discovered. Therefore, the assay developed here will be a useful tool for the discovery of new anti-influenza virus drug candidates.

Regarding its technical aspects, the HA inhibitor screening system developed here can provide more robust results than other assays, such as the plaque reduction assay and the hemagglutination inhibition assay, for screening antiviral drug candidates and HA inhibitors. Notably, other antiviral drug screening assays are performed on live host cells and related viruses, and as a result, these data are often impacted by the viability of host cell^31^,^32^. Specifically, in the plaque reduction assay, the size and number of plaques caused by viral infection are variable, and determining whether a given plaque should be counted is difficult. The hemagglutination inhibition assay used to screen HA inhibitors utilizes live erythrocytes and influenza viruses, and the inhibition of HA is determined according to the level of hemagglutination-producing complexes of influenza HA and the erythrocyte. However, the level of hemagglutination is assessed based on slight differences in reddish colour. Therefore, the plaque reduction and hemagglutination inhibition assays are semi-quantitative rather than quantitative methods. In contrast, our new *in vitro* HA inhibitor screening assay can be conducted using recombinant HA and does not require live host cells and influenza viruses. Therefore, the results of this assay are reproducible and do not depend on the viability of host cells. Additionally, the inhibitory activity of HA in this screening assay is precisely measured by changes in the FRET corresponding to the different states (docking, hemifusion, and full fusion), as in previous single-vesicle fusion assays^12^. Unlike the ensemble assays described above, this HA inhibitor screening assay can also measure the fusion of single vesicles individually. Therefore, this assay can provide robust results regarding HA inhibition by chemical species of interest.

In conclusion, similar to NA, HA plays a pivotal role in the life cycle of the influenza virus and is, therefore, an important druggable target protein for the treatment of influenza viruses that are resistant to NA inhibitors. Hence, the *in vitro* assay described here is a promising method to screen for HA inhibitors and can be used in Biosafety Level 1 facilities in addition to facilities at or above Biosafety Level 2.

## Additional Information

**How to cite this article**: Lee, H. *et al*. A new *in vitro* hemagglutinin inhibitor screening system based on a single-vesicle fusion assay. *Sci. Rep.* **6**, 30642; doi: 10.1038/srep30642 (2016).

## Supplementary Material

Supplementary Information
