# Supplementary material for: A new in vitro hemagglutinin inhibitor screening system based on a single-vesicle fusion assay
Source: Sci Rep. 2016 Jul 29;6:30642. doi: 10.1038/srep30642 (PMC4965830; doi:10.1038/srep30642)
Supplement: Supplementary Information [file srep30642-s1.pdf]

Supplementary information

A new in vitro hemagglutinin inhibitor screening system based on a single-vesicle fusion assay

Hanki Lee[1†,*], Wook Jin[3, 4†], Byeong-Chul Jeong[2] and Joo-Won Suh[1, 2*]

[1] Center for Nutraceutical and Pharmaceutical Materials, Myongji University, Yongin, Gyeonggi-do, 17058, Republic of Korea

[2] Division of Biosciences and Bioinformatics, College of Natural Science, Myongji University, Yongin, Gyeonggi-do, 17058, Republic of Korea

[3] Laboratory of Molecular Disease and Cell Regulation, Department of Molecular Medicine, School of Medicine, Gacheon University, Incheon, 21936, Republic of Korea

[4] Gachon Medical Research Institute, Gil Medical Center, Incheon, 21565, Republic of Korea

[†] These authors contributed equally to this study.

[*] Correspondence should be addressed to H. Lee (hklee@mju.ac.kr) or J.-W. Suh (jwsuh@mju.ac.kr).

This supplementary information contains

Supplementary Figures 1–5

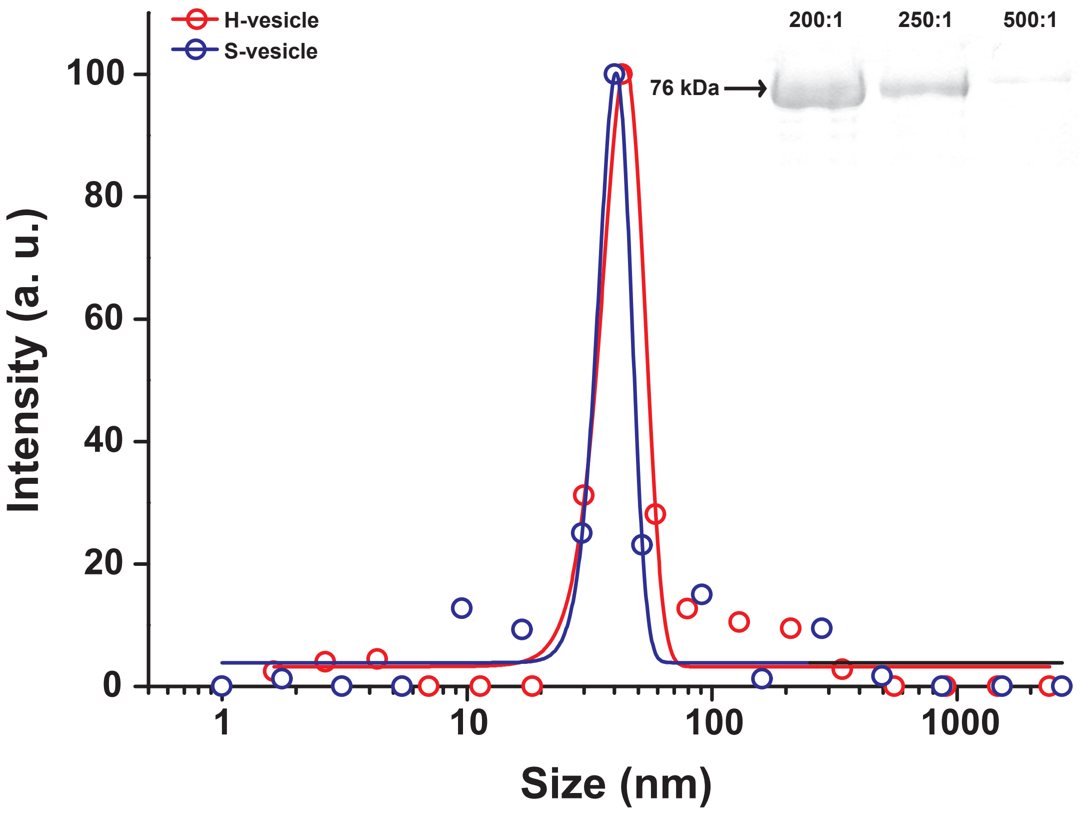

Supplementary Figure 1. The physicochemical characteristics of H- and S-vesicles. The sizes of H- and S-vesicles were fitted by a Gaussian algorithm. The inset figure indicates the level of HA incorporated into H-vesicles according to different ratios of lipid to HA, with HA bands visualised by silver staining and 10% SDS-PAGE.

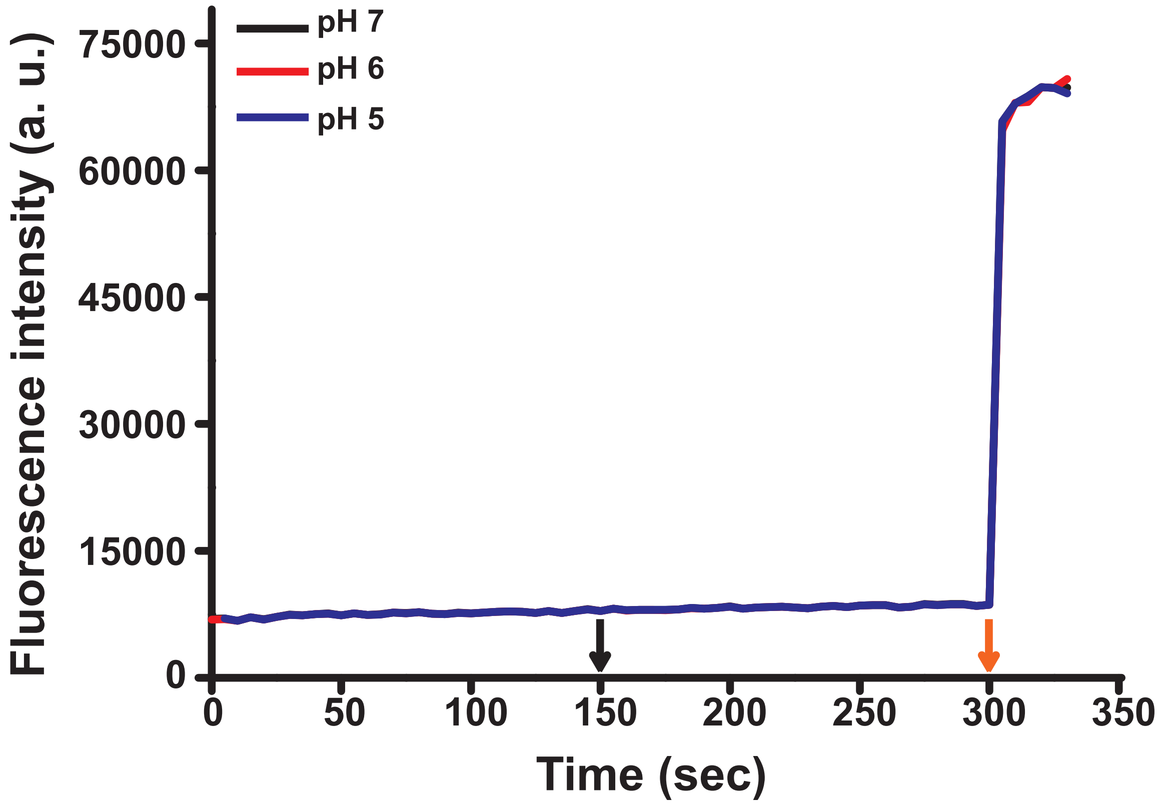

Supplementary Figure 2. The vesicle fusion with H-vesicles without HA and S-vesicle in the ensemble assay. The mixture with H-vesicles without HA and S-vesicles was incubated at room temperature in various pH conditions individually. The fluorescence intensity by the fusion between two different vesicles was monitored by the measurement of 665 nm fluorescent emission by the 549 nm excitation. The concentration of trypsin was 1% (w/v), which was an excessive amount for the complete digestion of HA in H-vesicles. Black and orange arrows indicate the time when trypsin and 1% Triton X-100 were treated, respectively.

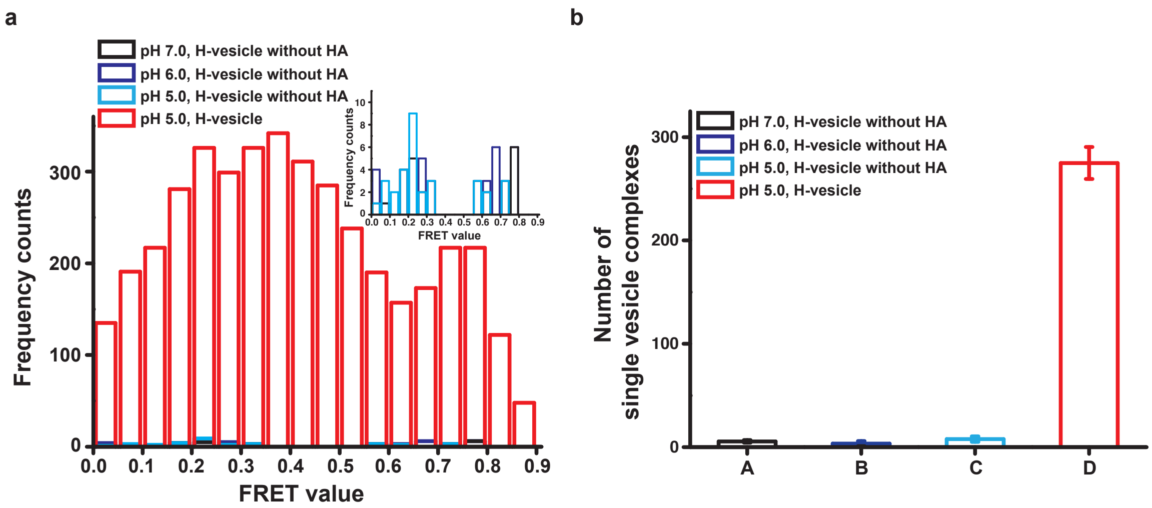

Supplementary Figure 3. The fusion with H-vesicles without HA and S-vesicles in the single vesicle fusion assay. (a) Fusion pattern of single vesicle complexes according to the interaction between H-without HA and S-vesicles. Red bar was revisited from Fig. 2. Inset only showed the fusion pattern with H-vesicles without HA and S-vesicles. (b) The number of single vesicle complexes according to the interaction between H- and S-vesicles. Red bar was revisited from Fig. 2. This experiment was carried out in triplicate.

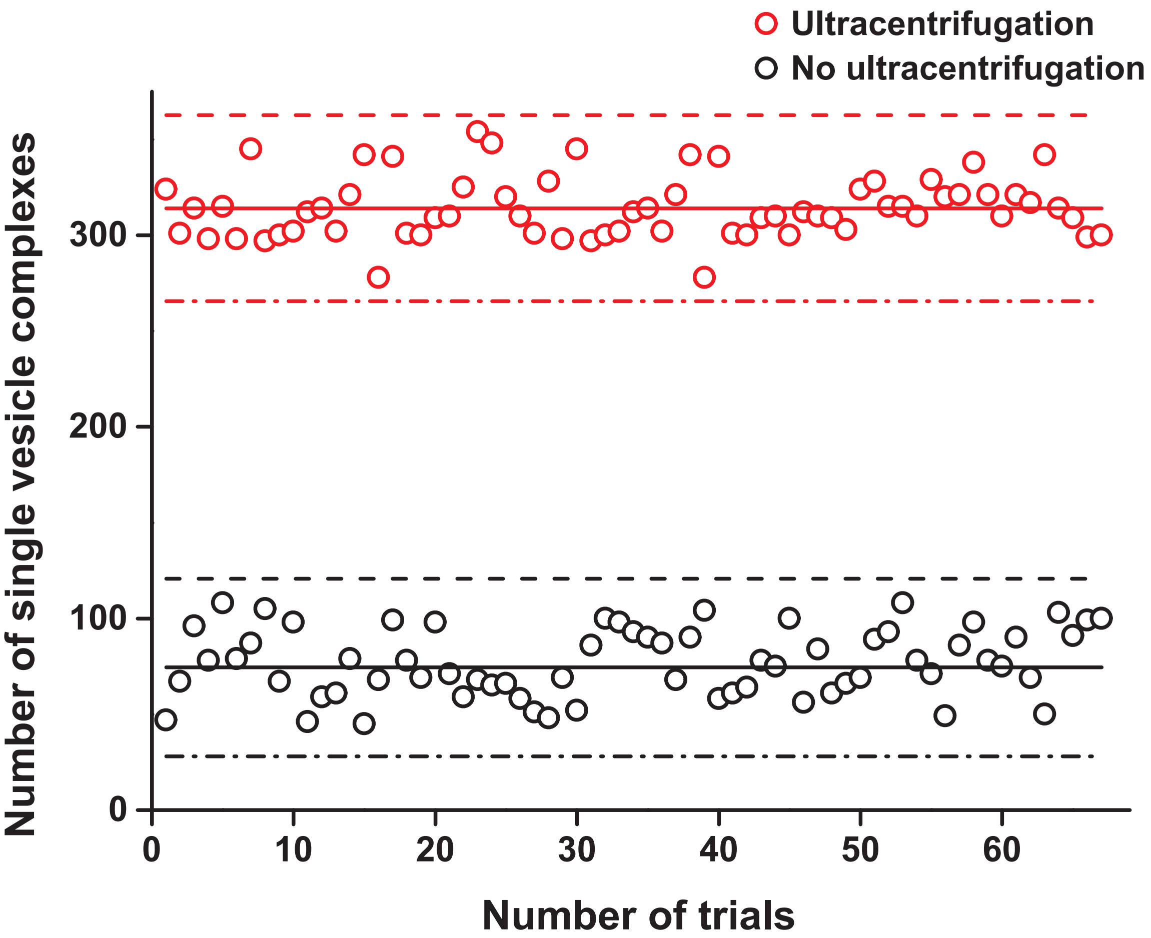

Supplementary Figure 4. The number of single vesicle complexes using H-vesicle according to the treatment of ultracentrifugation. This data was obtained from independent 67 experiments and all materials for the preparation of single vesicles was newly used in each 8th experiment. Line, dashed and dash-dotted line indicate the average, upper and lower limit of 3×SD (standard deviation) from each average value, respectively.

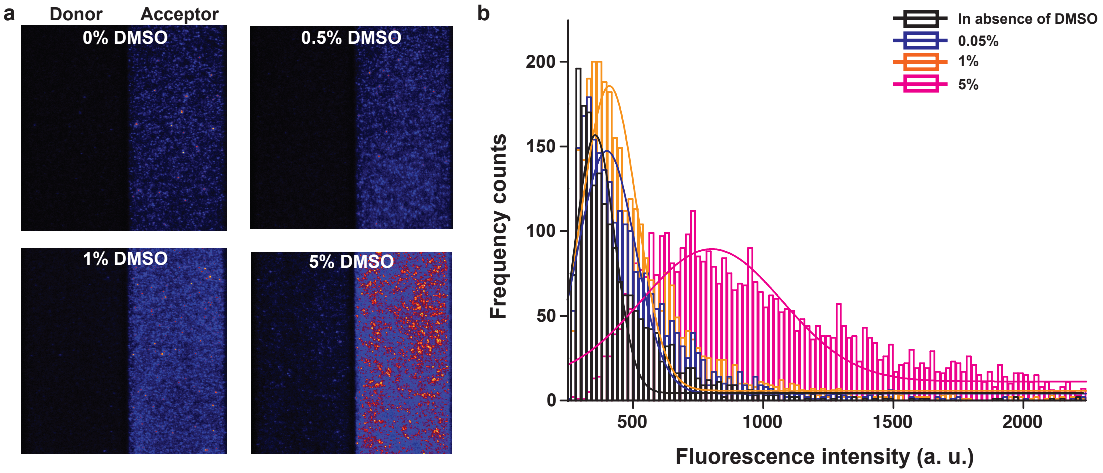

Supplementary Figure 5. The distortion of single vesicles according to the DMSO concentration. (a) Exemplary images of S-vesicles according to treatment with different DMSO concentrations. (b) Fluorescence distribution of S-vesicles according to treatment with different DMSO concentrations. The data were analysed through the captured images in (a) and fitted by a Gaussian algorithm.
